# Loss of a Guidewire During Central Venous Catheter Insertion Into the Right Internal Jugular Vein

**DOI:** 10.7759/cureus.51931

**Published:** 2024-01-09

**Authors:** Pedro Manuel Batarda Sena, Francisco Das Neves Coelho, Lourenço Castro Sousa

**Affiliations:** 1 Intensive Care Department, Hospital Central do Funchal, Funchal, PRT; 2 Helicopter Emergency Medical Service, Instituto Nacional de Emergencia Medica, Lisboa, PRT; 3 Intensive Care Department, Hospital Egas Moniz, Centro Hospitalar Lisboa Ocidental, Lisboa, PRT; 4 Vascular Surgery, Hospital Egas Moniz, Centro Hospitalar Lisboa Ocidental, Lisboa, PRT

**Keywords:** retained guidewire, iatrogenic complication, snare-assisted, intensive care unit, central venous access

## Abstract

Central venous catheter (CVC) insertion is a widely practiced technique for various purposes, such as administering fluids and medications, performing hemodialysis, and monitoring central venous pressure. During CVC insertion, numerous complications can arise, including the loss of the guidewire.

This case involves a 35-year-old female undergoing long-term hospitalization due to severe brain injury resulting from head trauma. She required long-term antibiotic therapy. Following the insertion of the CVC, the loss of the guidewire was noted, prompting immediate chest and abdominal X-rays, revealing its location in the inferior vena cava. The guidewire was surgically removed without any recorded complications.

To mitigate such complications, it is crucial to enhance technical proficiency in insertion techniques, seek supervision during procedures, and meticulously attend to every detail of the process, adhering closely to approved protocols or guidelines.

## Introduction

Central venous catheterization, a routine procedure in emergency and intensive care settings, serves critical functions such as fluid and medication administration, hemodialysis, blood and blood product transfusion, parenteral nutrition, and central venous pressure monitoring [1]. The Seldinger method is the recommended approach for placement due to its recognized safety. Despite its routine nature, the procedure is not without risks, and complications may arise in as many as 15% of these procedures [1,2]. Complications related to the guidewire are among these risks, holding the potential for serious outcomes and increased morbidity and mortality [1-4].

This article presents a case involving the loss of a guidewire during ultrasound-guided central venous catheter (CVC) insertion into the right internal jugular vein of a patient during her extended hospital stay. In this specific insertion site, approximately one-third of the wire enters directly into the inferior vena cava (IVC), as evidenced in the chest/abdominal X-rays [4]. The unique positioning enabled the safe extraction of the guidewire through an intravascular approach using an endovascular technique through the femoral vein, and this procedure was completed without immediate complications.

While occurrences of such events are rare, it remains crucial to recognize the diverse factors contributing to this type of complication [4]. Procedures conducted in urgent or emergency contexts demand heightened vigilance and meticulous care [5].

## Case presentation

The patient, a 35-year-old woman, underwent an extended hospital stay, which is a consequence of enduring severe head trauma resulting in a permanent injury. Due to long-term antibiotic therapy administration need, a 16-cm CVC was inserted into the right internal jugular vein, guided by ultrasound. 

**Figure 1:**
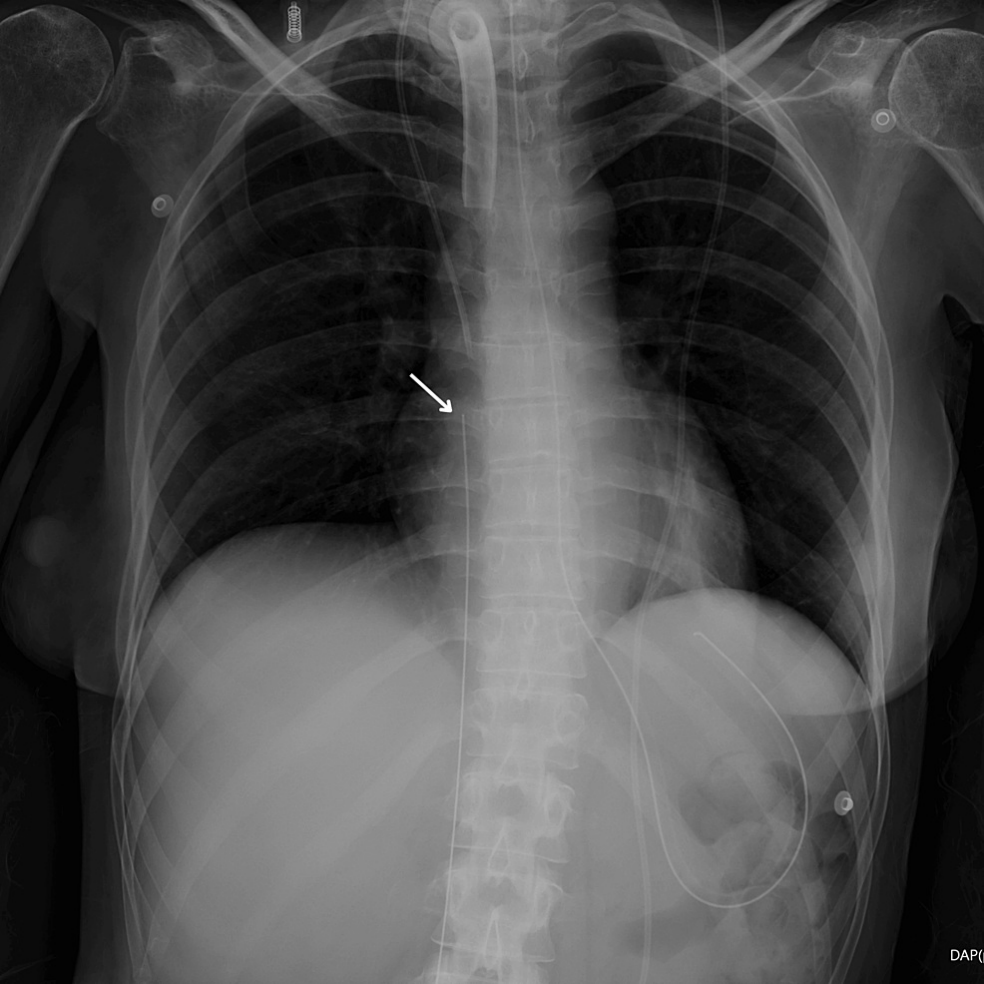
Chest X-ray The proximal part of the guidewire

**Figure 2:**
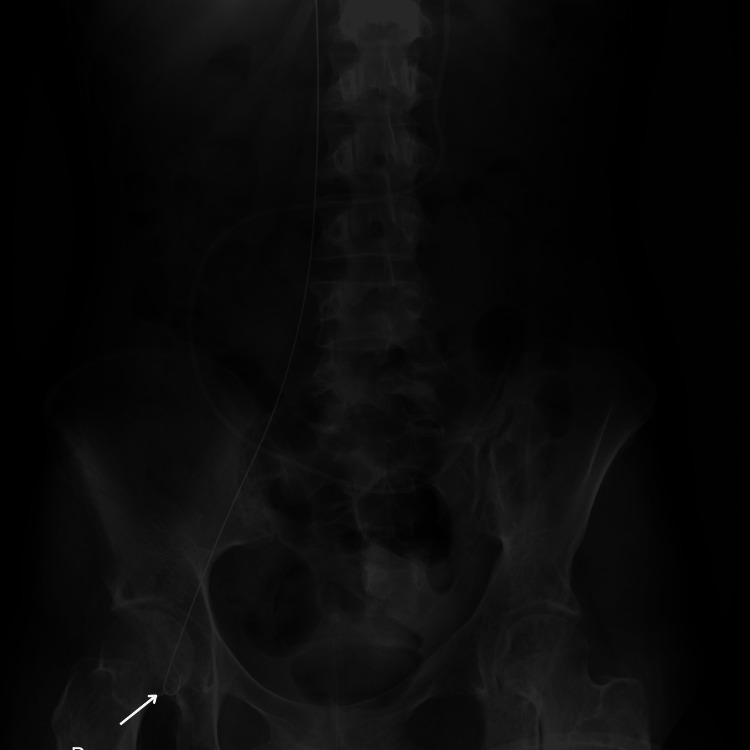
Abdominal X-ray The distal part of the guidewire

Our case, in which the guidewire insertion was performed by a resident experienced in central vascular accesses, underscores that such complications can arise even under the supervision of skilled personnel [5]. While the surgical procedure proceeded without other incidents, upon conducting an equipment check at the surgical table, the absence of the guidewire was noted. A point-of-care ultrasound was performed, which revealed the presence of the guidewire in the IVC and the absence of any cardiac complications. Chest and abdominal X-rays were immediately requested to document the position of the guidewire and its integrity (Figures 1, 2).

Taking into account the patient’s precarious clinical state, an interhospital transfer was considered excessively risky if the patient’s clinical situation could be promptly resolved within the hospital facilities. The surgical team considered the use of the N-Gage device, commonly used in urology for the removal of calculi, for guidewire extraction from the patient’s vascular system [1].

The N-Gage, typically featuring a wire basket for capturing and removing urinary tract stones, was repurposed to retrieve the guidewire from the IVC. Prior to this unconventional usage, and because of the lack of similar previously reported cases in the literature, an extensive evaluation of the ethical and safety implications was undertaken, which included a risk-benefit analysis of employing the N-Gage off-label and obtaining informed consent that detailed the non-standard nature of the impending procedure.

Meticulous planning preceded the adaptation of the N-Gage for vascular use. This involved ensuring the device’s compatibility with the venous system and sterility for intravascular application. The extraction procedure was closely monitored using fluoroscopy, facilitating precise tracking of the guidewire and the N-Gage. The team exercised exceptional caution during the manipulation of the N-Gage to avert vascular injury, given its original design for a different anatomical context.

Throughout the procedure, the patient’s vital signs were rigorously monitored, and the medical team was prepared to manage potential complications promptly. Following the successful retrieval of the guidewire, comprehensive post-procedure care, including monitoring and imaging exams, was conducted to confirm the absence of complications such as hemorrhage or embolism.

## Discussion

Inserting a CVC is a common procedure with inherent risks, one of which is the rare but significant complication of guidewire loss. The potential for this occurrence to cause serious adverse outcomes, such as arrhythmias, thrombosis, embolism, vessel perforation, and internal bleeding, cannot be underestimated [4,6].

To prevent such complications, a rigorous protocol must be followed. This should include the involvement of at least two physicians, one to perform the CVC insertion and another one to supervise and ensure that each step is meticulously performed. It is critical to maintain control of the guidewire’s tip throughout the procedure and to verify its complete removal once the procedure is concluded. The use of a detailed equipment checklist and conducting post-insertion imaging, such as chest and abdominal X-rays, are imperative practices. When reviewed collaboratively by the team, these tools serve as additional safeguards [6,7].

However, this case illustrates an instance where the guidewire was misplaced, despite these precautions. Due to the unavailability of standard retrieval tools like a snare during an off-hours period, the medical team had to improvise with the N-Gage device, a stone retrieval instrument commonly used in urology. This unconventional approach required the team to apply their in-depth knowledge of device mechanics and their creativity to safely adapt the tool for vascular use. The successful retrieval of the guidewire without any reported complications is a testament to the team’s adaptability and commitment to patient safety.

This case serves as a powerful testament to the necessity for ingenuity and resourcefulness in medical emergencies. It particularly showcases the surgeon’s quick thinking and interdisciplinary awareness by utilizing the N-Gage, a device from a different specialty, for successful guidewire retrieval. This decision, born from an acute understanding of the device’s functionality across specialties, exemplifies the importance of a broad knowledge base and the ability to apply it inventively in critical situations [6,7]

The incident reinforces the value of continuous multidisciplinary training and simulation to prepare for rare and unforeseen complications. Such preparation can enable medical professionals to act decisively and innovatively, ensuring patient safety when conventional tools and procedures are lacking.

Moreover, this episode of clinical ingenuity underscores the need for ongoing discussions regarding the ethical and safety implications of using non-standard equipment during emergencies. It stresses the importance of having a comprehensive understanding of various medical tools and the confidence to employ them creatively to achieve the best outcomes for patients, all while maintaining rigorous safety standards [4].

Ultimately, this case reinforces not only the need for strict adherence to protocols but also attention to detail in routine medical procedures.

Post-procedurally, this case necessitated an institutional review and reporting, contributing to policy development and enhanced preparedness for similar emergencies in the future. It also stresses the importance of comprehensive emergency improvisation training for healthcare professionals.

## Conclusions

This case serves as a critical reminder of the inherent complexities and potential risks involved in routine procedures like central venous catheterization. The occurrence of rare and preventable complications, such as guidewire loss, states the importance of a high level of vigilance and strict adherence to established protocols.

The successful resolution of this complication underscores the necessity for medical professionals to maintain flexibility and resourcefulness, especially when confronted with unforeseen procedural complications, and brings to light the ethical and safety considerations in employing non-standard equipment in emergency situations.
